# Approaches to interpreting and choosing the best treatments in network meta-analyses

**DOI:** 10.1186/s13643-017-0473-z

**Published:** 2017-04-12

**Authors:** L. Mbuagbaw, B. Rochwerg, R. Jaeschke, D. Heels-Andsell, W. Alhazzani, L. Thabane, Gordon H. Guyatt

**Affiliations:** 1 Department of Health Research Methods, Evidence and Impact, McMaster University, Hamilton, ON Canada; 2 Biostatistics Unit, Father Sean O’Sullivan Research Centre, St Joseph’s Healthcare, Hamilton, ON Canada; 3 Centre for Development of Best Practices in Health (CDBPH), Yaoundé Central Hospital, Yaoundé, Cameroon; 4 Department of Medicine, McMaster University, Hamilton, ON Canada; 5 Department of Paediatrics, McMaster University, Hamilton, ON Canada; 6 Centre for Evaluation of Medicine, St Joseph’s Healthcare—Hamilton, Hamilton, ON Canada; 7 Population Health Research Institute, Hamilton Health Sciences, Hamilton, ON Canada; 8 CLARITY Research Group, Department of Clinical Epidemiology & Biostatistics, McMaster University, Room 2C12, 1200 Main Street West, Hamilton, ON L8N 3Z5 Canada; 9 Department of Anaesthesia, McMaster University, Hamilton, ON Canada

**Keywords:** Ranking, SUCRA, Network meta-analysis, Advantages, Limitations

## Abstract

When randomized trials have addressed multiple interventions for the same health problem, network meta-analyses (NMAs) permit researchers to statistically pool data from individual studies including evidence from both direct and indirect comparisons. Grasping the significance of the results of NMAs may be very challenging. Authors may present the findings from such analyses in several numerical and graphical ways. In this paper, we discuss ranking strategies and visual depictions of rank, including the surface under the cumulative ranking (SUCRA) curve method. We present ranking approaches’ merits and limitations and provide an example of how to apply the results of a NMA to clinical practice.

## Background

Systematic reviews of randomized clinical trials (RCTs) provide crucial information for determining the effect of interventions in clinical practice [1]. Typically, investigators statistically combine treatment effect estimates (effect sizes) from individual clinical trials [2]. Traditional meta-analyses compare a single intervention to a single alternative (direct pair-wise comparisons) [3].

In many clinical contexts, clinicians consider more than two alternative treatments, each of which may have been compared to standard care, a placebo, or an alternative intervention. Because some interventions have never been compared to a placebo, or lack head-to-head direct comparisons, choosing between a number of alternatives creates challenges for determining their relative merit [4].

A solution to the multiple alternative problem that uses an entire body of evidence with all available direct and indirect comparisons—termed network meta-analysis (NMA) or multiple treatment comparison meta-analysis—is seeing increasing use [5]. In addition to providing information on the relative merits of interventions that have never been directly compared, NMAs may also increase the precision of effect estimates by combining both direct and indirect evidence.

However, the results of NMAs may be complex and difficult to interpret for clinicians especially when there are many alternative strategies and outcomes to consider [6, 7]. Guidance on how to interpret findings from NMAs remains limited [8]. To address interpretation challenges, NMA authors can complement numerical data with graphical tools [9–11] and by ranking interventions. Indeed, some form of ranking is reported in two thirds of all published NMAs [7], and experts recommend ranking as a form of presentation [12].

Other discussions have addressed reporting options, including ranking approaches, often assuming that readers have a sophisticated knowledge of analytic methods [9, 10]. Our objective here is not to be technical or comprehensive, but rather to discuss the merits and limitations of ranking methods with a specific focus on surface under the cumulative ranking (SUCRA) curve, a popular ranking method.

## Ranking treatments

Clinicians wish to offer patients a choice among the most desirable treatment options. Though a treatment that is certain to be the best in terms of the most important benefit outcome (e.g. a reduction in risk of stroke) would be a strong candidate for the treatment of choice, it might also carry more harms than other options (e.g. greatest risk of bleeding, or greatest burden).

Moreover, results of studies are always associated with uncertainty and we will seldom, if ever, be sure a treatment is best. Rather, we can think of the likelihood that, for a particular outcome, a treatment is best, or near best. Of two treatments that are unlikely to be the best, the treatment with a higher likelihood of being second best would—all else being equal—be preferable to one with a lower likelihood of being second best. Ranks can be presented graphically and numerically. The graphical approaches involve examining the area under the curve indicating the probability of each drug to occupy a specific rank. These graphs are daunting to compare, especially when many treatments and outcomes are examined.

The surface under the cumulative ranking curve (SUCRA) is a numeric presentation of the overall ranking and presents a single number associated with each treatment. SUCRA values range from 0 to 100%. The higher the SUCRA value, and the closer to 100%, the higher the likelihood that a therapy is in the top rank or one of the top ranks; the closer to 0 the SUCRA value, the more likely that a therapy is in the bottom rank, or one of the bottom ranks.

## Applying these methods to a real-life example

**Fig. 1:**
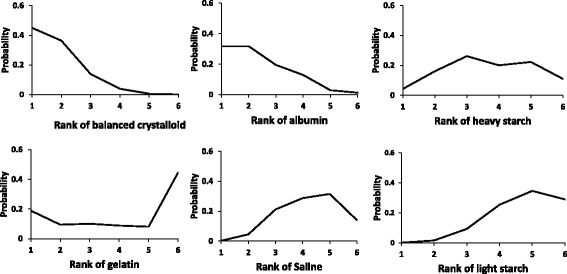
Graphical ranking of resuscitation fluids in six-node analysis

An NMA studied the impact of alternative resuscitative fluids on mortality in adult patients with sepsis [13]. We present here the results from an analysis that divided the intervention into six categories: albumin, balanced crystalloid, saline, gelatin, heavy starch and light starch. Figure 1 depicts the rankings of these six treatments.

From Fig. 1, we can see that balanced crystalloids have the highest likelihood of being ranked first, followed by albumin, gelatin and heavy starch; the results suggest no possibility that light starch and saline lead to the lowest mortality. For the second rank, balanced crystalloids and albumin still appear most likely and light starch and saline least likely, but heavy starch now has a higher likelihood than gelatin. Gelatin, the two starches, and saline are more likely to be among the lower ranks (3 to 6), and albumin and balanced crystalloid far less likely to be among the lower ranks. Looking across the figures, you could make an intuitive estimate of the rankings, and the gradient in effect across treatments.

Table 1 presents the SUCRA results that emerge from these data. The SUCRA rankings confirm that balanced crystalloid and albumin are most likely to result in the lowest mortality (with quite similar SUCRA scores) while light starch appears appreciably less attractive than the other alternatives

**Table 1:** SUCRA rankings from six-node analysis

Rank	Treatment	SUCRA
1	Balanced crystalloid	84.1%
2	Albumin	74.5%
3	Heavy starch	45.4%
4	Gelatin	37.7%
5	Saline	34.2%
6	Light starch	24.0%

*SUCRA* surface under the cumulative ranking curve

.

## Five reasons why these rankings may mislead if not interpreted correctly

Taking these results at surface value, clinicians should now be resuscitating all their septic patients with a balanced crystalloid solution. There are, however, several reasons why clinicians should not routinely choose a treatment with the higher SUCRA ranking. First, the evidence on which the SUCRA rankings are based may be of very low quality (synonyms: low certainty or confidence) and therefore untrustworthy. Second, there are typically several relevant outcomes. A treatment that is best in one outcome (say, a benefit outcome) may be the worst in another outcome (for example, a harm outcome). Third, issues such as cost and a clinician’s familiarity with use of a particular treatment may also bear consideration. Fourth, in the process of calculation, SUCRA does not consider the magnitude of differences in effects between treatments (e.g. in a particular simulation the first ranked treatment may be only slightly, or a great deal better than the second ranked treatment). Fifth, chance may explain any apparent difference between treatments, and SUCRA does not capture that possibility.

In this case, clinicians may easily misinterpret the apparently clear hierarchy in the efficacy of these fluids in reducing mortality. Table 2 presents a more detailed summary of the evidence, including the number of direct comparisons, the direct, indirect and network estimates and their associated credible intervals, and the certainty (quality, confidence) of the evidence.

**Table 2:** NMA results including certainty assessments

Comparison	Number of trials with direct comparisons	Direct estimate (95% CI)	Indirect estimate (95% CrI)	NMA estimate (95% CrI) (higher of direct or indirect confidence)
Light starch vs saline	4	1.07 (0.89, 1.29) M^1^	0.59 (0.25, 1.35) VL^1,2,3^	1.04 (0.87, 1.25) M
Heavy starch vs saline	3	0.64 (0.30, 1.37) M^1^	1.13 (0.71, 1.80) VL^1,2^	0.95 (0.64, 1.41) M
Albumin vs saline	2	0.81 (0.64, 1.03) M^1^	0.96 (0.14, 6.31) VL^2,4^	0.82 (0.65, 1.04) M
Balanced crystalloid vs saline	0	–	0.78 (0.58, 1.05) L^1,2^	0.78 (0.58, 1.05) L
Gelatin vs saline	0	–	1.04 (0.46, 2.32) VL^1,2^	1.04 (0.46, 2.32) VL
Heavy starch vs light starch	0	–	0.91 (0.63, 1.33) L^1,2^	0.91 (0.63, 1.33) L
Albumin vs light starch	0	–	0.79 (0.59, 1.06) L^1,2^	0.79 (0.59, 1.06) L
Balanced crystalloid vs light starch	2	0.80 (0.61, 1.04) M^3^	0.44 (0.19, 0.97) M^2^	0.75 (0.58, 0.97) M
Gelatin vs light starch	0	–	1.00 (0.44, 2.21) VL^1,2^	1.00 (0.44, 2.21) VL
Albumin vs heavy starch	2	1.40 (0.35, 5.56) L^4^	0.83 (0.52, 1.33) L^1,2^	0.87 (0.55, 1.36) L
Balanced crystalloid vs heavy starch	1	0.74 (0.52, 1.05) M^1^	1.35 (0.63, 2.92) VL^2,4^	0.82 (0.60, 1.13) M
Gelatin vs heavy starch	1	1.09 (0.55, 2.19) L^4^	–	1.10 (0.54, 2.21) L
Balanced crystalloid vs albumin	0	–	0.95 (0.65, 1.38) VL^1,2^	0.95 (0.65, 1.38) VL
Gelatin vs albumin	0	–	1.26 (0.55, 2.90) VL^2,4^	1.26 (0.55, 2.90) VL
Gelatin vs balanced crystalloid	0	–	1.34 (0.61, 2.89) VL^2,4^	1.34 (0.61, 2.89) VL

“From Annals of Internal Medicine, Rochwerg B et al, Fluid Resuscitation in Sepsis: A systematic review and network meta-analysis, 161, 5, 347-55.”

*CI* confidence interval, *CrI* credible interval; QoE: *H* high, *M* moderate, *L* low, *VL* very low

^1^—rated down for imprecision, ^2^—rated down for indirectness, ^3^—rated down for inconsistency (*I* ^2^ = 80%, *p* = 0.03 for heterogeneity), ^4^—rated down 2 levels for imprecision

This body of evidence demonstrates the most compelling reason to potentially mistrust rankings in general and SUCRA in particular: they may arise from evidence warranting low or very low certainty. A set of SUCRA ratings may arise from a large body of studies with few limitations and high certainty in the evidence. Exactly the same set of ratings may arise from a small body of studies with major limitations in risk of bias (unconcealed randomization, lack of blinding, large loss to follow-up), imprecision (wide confidence intervals or small number of events), inconsistency in results, indirectness (for instance, studies enrolling a sample of patients that differ from the population of interest, or measuring outcomes differently, such as with shorter follow-up), and publication bias—and thus warrant only low or very low certainty.

In this case, because of a high risk of bias, imprecision, inconsistency, and indirectness, of the 15 paired comparisons, 5 warrant only very low certainty, 5 low certainty, 5 moderate certainty, and none high certainty. Of the moderate certainty comparisons, only 1, balanced crystalloid versus low starch, showed a statistically significant (i.e. *p <* 0.05) difference between treatments; all the other moderate certainty ratings failed to show a statistically significant difference between treatments (indeed, none of the other 10 paired comparisons showed convincing differences either).

Because of the low or very low quality evidence underlying most comparisons, the SUCRA ratings will result in misleading inferences if taken at face value. For instance, we may reasonably infer from Table 2 that balanced crystalloids are very likely to result in lower mortality than light starch. We cannot be at all certain, however, that the differences between balanced crystalloid and albumin, or even balanced crystalloid and heavy starch, are real and important. Indeed, and perhaps wisely, reviewers of the NMA felt that the risk of misinterpretation of rankings in general and SUCRA in particular was in this case so great that they insisted on their omission from the published manuscript [13]. However, most clinicians are likely to find interpretation of Table 2 data challenging. Indeed, this is likely to be the case whenever an NMA includes more than three or four interventions. Therefore, despite their limitations, alternative presentation formats are likely to be helpful.

## An alternative summary presentation

Given the risks of relying primarily on rankings, and the cognitive challenges of processing tabular presentations such as Table 2 (which has the benefit of capturing all the key evidence), there is another potentially helpful presentation format for NMAs. This format involves a visual representation of point estimates and certainty or confidence intervals comparing NMA estimates of each treatment against a constant comparator. In NMAs comparing alternative drug therapies, that common comparator may be a placebo or standard care.

**Fig. 2:**
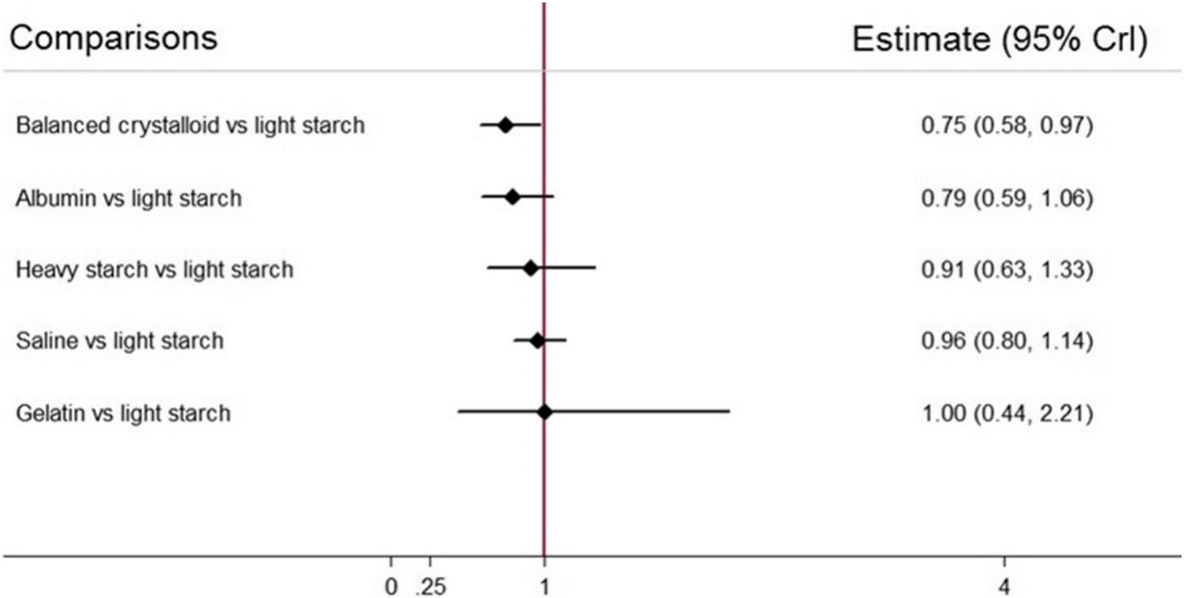
Point estimates and confidence intervals of comparisons between the five alternative resuscitation fluids and light starch

In this case, we have chosen the lowest ranked treatment, light starch (Fig. 2) as the common comparator. This visual representation facilitates appropriate inferences: (i) point estimates suggest that all treatments (with the exception of gelatin, with a point estimate of 1.0) are superior to light starch; (ii) any true differences between balanced crystalloid and albumen are likely to be small; (iii) differences between these two treatments and the other four may be considerably larger and (iv) the extent of the overlapping confidence intervals considerably diminishes our certainty about inferences (i) to (iii).

While potentially helpful, and in particular at least to some extent avoiding the excessively strong inferences that the unwary clinician might make from SUCRA rankings, this presentation format also has limitations. First, it deals only with a few of the comparisons in Table 2, and a full picture of the evidence requires a consideration of the other comparisons. Second, while capturing issues of precision, it tells us nothing about risk of bias, indirectness, and publication bias, and a limited amount about inconsistency (if the analysis is based on random—rather than fixed—effect models, inconsistency may contribute to widening of confidence intervals). Third, using a common comparator to which many interventions have not been compared may lead to wider confidence intervals, leading to less secure inferences than the data may warrant.

However, in our current example, all else being equal, the evidence from the visual display of rankings, from the SUCRA ratings, and from the visual depiction of comparisons with light starch all suggest that choosing either balanced crystalloid or albumin as the initial resuscitation fluid may be advisable. At least one inference is very secure: light starch is a poor choice of resuscitation fluid.

## Conclusions

We acknowledge some limitations in this work. Our descriptions are based on one example in which the differences between the effects of the resuscitation fluids is not very large, and therefore careful consideration is required in selecting the best option.

Appropriate interpretation of NMA results involves presentation of direct and indirect as well as the NMA estimates and their associated confidence/credible intervals for each paired comparison, as well as the associated certainty of estimates (as in Table 2). When the NMA involves more than three or four interventions; however, the cognitive challenge of optimally interpreting such evidence summaries is daunting. Visual displays of rankings (Fig. 1), the SUCRA statistic (Table 1), and visual displays of point estimates and confidence intervals of relative effects of interventions against a common comparator (Fig. 2) can all aid in interpretation when used together.

Clinicians using NMAs should bear in mind that the presentation approaches we have described all have their limitations and require cautious interpretation. If interpreted in the light of certainty (quality and confidence) in the evidence, clinicians can avoid misleading inferences. They can then use best evidence presentations from NMA to guide their clinical practice and offer patients optimal choices in managing their health issues.

## Abbreviations

NMA: Network meta-analysis
SUCRA: Surface under the cumulative ranking
